# Clustering of glycoprotein VI (GPVI) dimers upon adhesion to collagen as a mechanism to regulate GPVI signaling in platelets

**DOI:** 10.1111/jth.13613

**Published:** 2017-02-16

**Authors:** N. S. Poulter, A. Y. Pollitt, D. M. Owen, E. E. Gardiner, R. K. Andrews, H. Shimizu, D. Ishikawa, D. Bihan, R. W. Farndale, M. Moroi, S. P. Watson, S. M. Jung

**Affiliations:** ^1^ Institute of Cardiovascular SciencesCollege of Medical and Dental SciencesUniversity of BirminghamBirminghamUK; ^2^ Department of Physics and Randall Division of Cell and Molecular BiophysicsKing's College LondonLondonUK; ^3^ Department of Cancer Biology and TherapeuticsJohn Curtin School of Medical ResearchAustralian National UniversityCanberraACTAustralia; ^4^ Australian Centre for Blood DiseasesMonash UniversityMelbourneVictoriaAustralia; ^5^ Research DepartmentChemo‐Sero‐Therapeutic Research InstituteKaketsukenKumamotoJapan; ^6^ Department of BiochemistryUniversity of CambridgeCambridgeUK; ^7^ Centre for Membrane Proteins and Receptors (COMPARE)College of Medical and Dental SciencesUniversity of BirminghamBirminghamUK; ^8^ Present address: Institute for Cardiovascular and Metabolic ResearchSchool of Biological SciencesUniversity of ReadingReadingRG6 6ASUK

**Keywords:** glycoprotein, platelet activation, platelet adhesiveness, platelet membrane glycoproteins, receptors, collagen

## Abstract

**Essentials**

- Dimeric high‐affinity collagen receptor glycoprotein VI (GPVI) is present on resting platelets.
- Spatio‐temporal organization of platelet GPVI‐dimers was evaluated using advanced microscopy.
- Upon platelet adhesion to collagenous substrates, GPVI‐dimers coalesce to form clusters.
- Clustering of GPVI‐dimers may increase avidity and facilitate platelet activation

**Summary:**

**Background**

Platelet glycoprotein VI (GPVI) binding to subendothelial collagen exposed upon blood vessel injury initiates thrombus formation. Dimeric GPVI has high affinity for collagen, and occurs constitutively on resting platelets.

**Objective**

To identify higher‐order oligomerization (clustering) of pre‐existing GPVI dimers upon interaction with collagen as a mechanism to initiate GPVI‐mediated signaling.

**Methods**

GPVI was located by use of fluorophore‐conjugated GPVI dimer‐specific Fab (antigen‐binding fragment). The tested substrates include Horm collagen I fibers, soluble collagen III, GPVI‐specific collagen peptides, and fibrinogen. GPVI dimer clusters on the platelet surface interacting with these substrates were visualized with complementary imaging techniques: total internal reflection fluorescence microscopy to monitor real‐time interactions, and direct stochastic optical reconstruction microscopy (dSTORM), providing relative quantification of GPVI cluster size and density. Confocal microscopy was used to locate GPVI dimer clusters, glycoprotein Ib, integrin α_2_β_1_, and phosphotyrosine.

**Results**

Upon platelet adhesion to all collagenous substrates, GPVI dimers coalesced to form clusters; notably clusters formed along the fibers of Horm collagen. dSTORM revealed that GPVI density within clusters depended on the substrate, collagen III being the most effective. Clusters on fibrinogen‐adhered platelets were much smaller and more numerous; whether these are pre‐existing oligomers of GPVI dimers or fibrinogen‐induced is not clear. Some GPVI dimer clusters colocalized with areas of phosphotyrosine, indicative of signaling activity. Integrin α_2_β_1_ was localized to collagen fibers close to GPVI dimer clusters. GPVI clustering depends on a dynamic actin cytoskeleton.

**Conclusions**

Platelet adhesion to collagen induces GPVI dimer clustering. GPVI clustering increases both avidity for collagen and the proximity of GPVI‐associated signaling molecules, which may be crucial for the initiation and persistence of signaling.

## Introduction

Upon blood vessel injury, circulating platelets interact with exposed subendothelial collagen through the collagen receptor glycoprotein VI (GPVI). This 65‐kDa immune receptor signals through its associated Fc‐receptor γ‐chain, which contains an immunoreceptor tyrosine‐based activation motif (ITAM) in its intracellular domain. Binding of GPVI to collagen induces phosphorylation of the ITAM residues, which can then bind to Syk, which itself becomes phosphorylated and activated. This process initiates signalosome assembly 1, leading to a series of downstream signals, resulting in platelet activation, finally culminating in thrombus formation.

GPVI contains two extracellular Ig‐like domains: D1, containing the collagen‐binding site 2, 3; and D2, connected via an O‐glycosylated stem to its transmembrane domain and short cytoplasmic tail 4. GPVI binds to tandem glycine–proline–hydroxyproline (GPO) sequences in collagen 5, 6. Surface plasmon resonance showed that dimerized recombinant GPVI (D1D2‐Fc)_2_ bound collagen fibers with high affinity, but binding of its monomeric form (D1D2) was too low to be measured 7. Monomeric and dimeric recombinant D1D2 showed similar affinities for collagen‐related peptide (CRP), a triple‐helical peptide containing 10 contiguous GPO triplets, suggesting that GPVI dimers may have a specific conformation that recognizes the higher‐order structure of fibrous collagen, beyond simply the GPO sequences. The crystal structure of a D1D2 dimeric assembly 8 allowed docking simulations 3, 5, 8, which suggested that D1 contained grooves large enough to accommodate the triple‐helical CRP. In 2009, Jung *et al*. provided direct evidence for the presence of dimers on the resting platelet surface with GPVI dimer‐specific, inhibitory m‐Fab‐F 9. Later, they reported a non‐inhibitory Fab, 204‐11, which recognized GPVI dimers 10, and used it to show that GPVI dimers were constitutively present on resting platelets. These observations suggested that the first interaction in collagen‐induced activation of platelets is collagen binding to GPVI dimers. Several groups, however, reported that platelet activation induced the formation of GPVI dimers. Arthur *et al*. 11 provided biochemical evidence for disulfide‐linked dimers in activated platelets. Loyau *et al*., employing GPVI dimer‐specific mAb 9E18, reported that GPVI dimerization was induced by soluble agonists or von Willebrand factor (VWF), with almost no dimers being detected on resting platelets 12, leading them to propose dimer formation as a means to control collagen‐induced platelet activation. Dimerization is an accepted mechanism for cell activation through receptor tyrosine kinases 13, whereby ligand binding to the receptor extracellular domains induces dimerization, causing a conformational change that brings together the kinase domains in the cytoplasmic tails, facilitating autophosphorylation, and thereby initiating intracellular signals. The Src‐family kinase Lyn associates with the cytoplasmic tail of GPVI 14, which lacks intrinsic kinase activity, so that FcRγ attached to one GPVI monomer might be phosphorylated by Lyn associated with a second monomer, brought into proximity by dimerization.

An alternative activation mechanism would be higher‐order receptor clustering 15, which does not preclude the presence of constitutive GPVI dimers in non‐activated platelets. Clustering has been demonstrated for many classes of receptor, including G‐protein‐coupled receptors 16, adhesion receptors such as platelet integrin α_IIb_β_3_ 17, platelet C‐type lectin‐like receptor 2 18, and, notably, discoidin domain receptor 1, a constitutively dimeric tyrosine kinase receptor for collagen 19.

As GPVI dimers are present on resting platelets, we hypothesize that a further level of control is required. We hypothesize that clustering of GPVI dimers is a plausible mechanism to modulate platelet activation once the high‐affinity, but low‐copy‐number, GPVI dimers engage the limited number of binding sites on collagen 2, 20. The formation of GPVI clusters could result in increased avidity and may bring associated signaling molecules together to facilitate platelet activation. We used complementary imaging techniques to test this hypothesis: total internal reflection fluorescence microscopy (TIRFM), to visualize in real time GPVI dimer distribution at the interface between the platelet membrane and immobilized collagenous substrates; direct stochastic optical reconstruction microscopy (dSTORM) for relative quantification of GPVI cluster size and density; and confocal microscopy to compare the localization of GPVI dimers and two other receptors involved in the platelet–collagen interaction, i.e. glycoprotein Ib (GPIb) and α_2_β_1_, and whether signaling was associated with the clustered GPVI dimers.

## Materials and methods

Non‐inhibitory, recombinant dimer‐specific 204‐11 Fab 10 was derived from clone 204‐11 21 by Kaketsuken (Kumamoto, Japan). Antibodies were fluorescently labeled by use of a Microscale Protein Labelling kit (Molecular Probes, Eugene, OR, USA); degree of fluorescent labeling = 2–5 dye molecules per protein molecule. The antibodies used were: 4G10 (anti‐phosphotyrosine; Millipore Merck, Billerica, MA, USA); anti‐human CD42b/GPIb, clone 486805 (R&D Systems, Abingdon, UK); 16B4 (mouse anti‐human CD49b/integrin α_2_ chain; Bio‐Rad, Hercules, CA, USA); Alexa Fluor 647‐conjugated AffiniPure F(ab′)2 fragment goat anti‐mouse IgG, Fcγ‐specific (Jackson ImmunoResearch Laboratories, Inc., West Grove, PA, USA); Gi9 (anti‐integrin α_2_; Abcam, Cambridge, UK); and Alexa Fluor 647‐conjugated anti‐human CD62P (Bio‐Rad). Other materials, of reagent grade or better, were obtained from commercial sources.

### Platelet preparation

Washed platelets were prepared from acid–citrate–dextrose‐anticoagulated blood from healthy volunteers 22, and resuspended at 3–5 × 10^7^ platelets mL^−1^ in HEPES–Tyrodes buffer (HT) (134 mm NaCl, 0.34 mm Na_2_HPO_4_, 2.9 mm KCl, 12 mm NaHCO_3_, 20 mm HEPES, 5 mm glucose, pH 7.3).

### Preparation of collagenous substrate‐coated glass dishes for imaging

Thirty‐five‐millimeter glass (0.7 mm)‐bottomed MatTek dishes (MatTek, Ashland, MA, USA) were coated with 10 μg mL^−1^ cross‐linked CRP (CRP‐XL), III‐30, or collagen III (Col III), in phosphate‐buffered saline (PBS) (0.01 m phosphate buffer, 0.0027 m KCl, 0.137 m NaCl, pH 7.4) and Horm collagen (Horm), in manufacturer‐supplied diluent, overnight at 4 °C. The dishes were washed with PBS, blocked with 1% bovine serum albumin/PBS (heat‐denatured, filtered) for 1 h, and then washed with PBS to make them ready for platelet spreading.

### TIRFM

Adhesion of Alexa Fluor 488–204‐11 Fab‐labeled washed platelets (3 × 10^7^ platelets mL^−1^ in HT containing 2 mm MgCl_2_) to immobilized collagenous substrate was imaged with TIRFM (Nikon TIRF system mounted on a Nikon Eclipse Ti inverted microscope, with a Nikon × 60 numerical aperture [NA] 1.49 TIRF objective; Nikon UK, Ltd., Surrey, UK). Images were obtained at 5‐s intervals for 20–30 min at 37 °C, and this was followed by fixation in formalin and confocal imaging. Integrin α_2_β_1_ blockade was achieved by preincubating platelets with 10 μg mL^−1^ Gi9 blocking antibody. Platelet morphology was followed with differential interference contrast (DIC) microscopy.

### dSTORM

Alexa Fluor 647‐204‐11 Fab‐labeled washed platelets were allowed to adhere to collagenous substrate‐coated MatTek dishes. Adherent platelets were fixed, permeabilized, and stained with phalloidin–Alexa Fluor 488. Samples were imaged in switching buffer as described previously 22 on a Nikon Eclipse Ti‐E N‐STORM system in dSTORM mode using Perfect Focus, with a CFl SR Apochromat TIRF × 100 oil, 1.49‐NA objective lens and an N‐STORM filter cube with excitation from the Agilent Ultra High Power Dual Output Laser bed (170‐mW, 647‐nm laser), and image capture with an Andor IXON Ultra 897 EMCCD camera. Thirty thousand frames were captured with Nikon nis elements v4.2, and reconstructed by the use of storm analysis module v3.2, with drift correction and Gaussian rendering of data points. Detected points with a photon count of < 500 were discarded from reconstructed data before further processing. Points in the reconstructed images represent individually identified fluorescent blinking events, which are referred to as molecules.

### Confocal imaging

Platelets were preincubated with Alexa Fluor 488‐conjugated or Alexa Fluor 647‐conjugated 204‐11 Fab (4 μg mL^−1^) alone or with another fluorescently labeled antibody. In some experiments, platelets were also treated with an inhibitor or inhibitory antibody. The platelets were allowed to adhere to a collagenous substrate, fixed, and imaged with an FV300 IX81 laser‐scanning confocal microscope with a × 60 oil immersion objective (Olympus UK, Southend‐on‐Sea, UK). Where indicated, platelets were permeabilized (0.1% Triton/PBS) following fixation, and stained for actin (Alexa Fluor 647–phalloidin) or phosphotyrosine (4G10, 5 μg mL^−1^).

### Flow cytometry to measure GPVI dimer formation

Washed platelets were preincubated with dimethylsulfoxide (DMSO) (0.25% final concentration) or actin antagonist (cytochalasin D, latrunculin A, or jasplakinolide; 10 μm; 0.25% DMSO, final concentration), and GPVI dimer formation was measured by flow cytometry 10.

### Data analyses

Data analyses, performed with prism v7 (GraphPad, San Diego, CA, USA), are described in the figure legends. Differences among treatment groups in the flow cytometry experiments were calculated with paired *t*‐tests. dSTORM cluster analysis was performed within matlab for each 3 × 3‐μm region of interest (ROI), as described by Owen *et al*. 23, with modifications described in Pollitt *et al*. 18. Platelets were identified by phalloidin staining of the actin cytoskeleton. ROIs were positioned within the platelet, ensuring that the edges of the ROI fell within the platelet boundary. Clusters of < 3 points were discounted. The numerical data, processed with Microsoft Excel, were analyzed as detailed in the figure legends. The degree of colocalization between the GPVI and phophotyrosine confocal images was quantified with image‐pro premiere 9.2 (Media Cybernetics, Rockville, MD, USA).

## Results

### TIRFM imaging of GPVI dimer cluster formation in live platelets interacting with immobilized collagenous substrates

**Figure 1:**
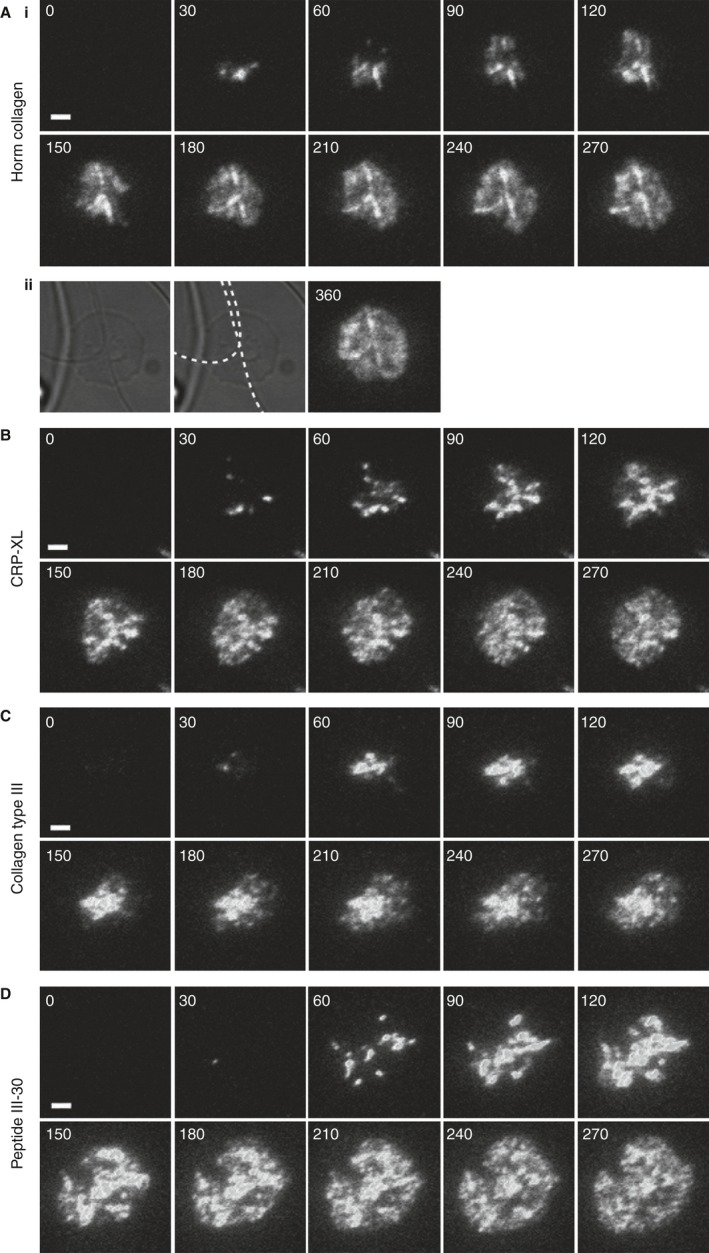
Glycoprotein VI forms clusters when platelets spread on immobilized collagenous substrates. Total internal reflection fluorescence microscopy time courses of washed human platelets, labeled with Alexa Fluor 488‐conjugated, dimer‐specific 204‐11 Fab, interacting with 10 μg mL ^−1^ immobilized collagenous substrates at 37 °C, are shown. (Ai) Horm collagen. (B) Cross‐linked collagen‐related peptide (CRP‐XL). (C) Non‐fibrous collagen type III. (D) Peptide III‐30. The positions of Horm collagen fibers are indicated on a differential interference contrast image by the dashed lines (Aii). Images are of a single platelet representative of three independent experiments. See Movie S1. Time stamp: seconds. Scale bar: 2 μm. [Color figure can be viewed at wileyonlinelibrary.com]

We investigated the spatial and temporal distribution of GPVI dimers on the surfaces of platelets interacting with different immobilized collagenous substrates (Table 1). Washed platelets labeled for GPVI dimer were allowed to adhere, and cluster formation and dynamics were visualized with TIRFM (Fig. 1; Movie S1). Discrete clustered GPVI dimers can be visualized in spreading platelets, whereas non‐clustered GPVI dimers are poorly resolved, appearing as diffuse fluorescence over the platelet surface.

**Table 1:** Collagenous substrates used in this study

Collagenous substrate	Abbreviation	Description	Structure^a^	Collagen receptor specificity
Cross‐linked collagen‐related peptide^b^	CRP‐XL	Triple‐helical, crosslinked	GCO‐(**GPO**)_10_GCOG‐NH_2_	GPVI
Collagen Toolkit III peptide 30^c^	III‐30	Triple‐helical	GPC‐(GPP)_5_‐GAOGARGGA‐**GPO**GPEGGKGAA**GPOGPO‐**(GPP)_5_‐GPC‐NH_2_	GPVI
Horm collagen (equine, type I)^d^	Horm	Fibrous Type I	[α_1_(I)]_2_α_2_(I)	GPVI Integrin α_2_β_1_
Bovine collagen type I^e^	Col I	Non‐fibrous	[α_1_(I)]_2_α_2_(I)	GPVI Integrin α_2_β_1_
Bovine collagen type III^e^	Col III	Non‐fibrous	[α_1_(III)]_3_	GPVI Integrin α_2_β_1_

a GPVI‐binding GPO triplets are shown in bold type for CRP and III‐30.

b CRP‐XL was synthesized as described by Morton *et al*. 29.

c Triple‐helical peptide III‐30 is from Collagen Toolkit III, a set of overlapping triple‐helical peptides encompassing the entire collagen domain of human collagen III; it was synthesized as described by Raynal *et al*. 30.

d Nycomed Pharma (1 mg mL^−1^; Munich, Germany).

e Koken (3 mg mL^−1^; Tokyo, Japan).

GPVI dimers formed clusters on platelets adhered to all collagenous substrates tested. Cluster formation on fibrous Horm followed a distinct distribution, being concentrated along the collagen fiber (Fig. 1Ai,ii), initially forming at the point of contact between the platelet and the fiber, and then propagating along the fiber (Fig. 1Aii). Clusters were not restricted to the visible collagen fiber, but were also present on the platelet surface, presumably because of indirect signal‐induced clustering (e.g. outside‐in signaling through released substances such as fibrinogen, ADP, and VWF), through contact with smaller (non‐visible) fibers in these portions of the surface, or both. The non‐fibrous collagenous substrates CRP‐XL (Fig. 1B), Col III (Fig. 1C) and III‐30 (Fig. 1D) also supported the formation of GPVI dimer clusters, each with similar cluster distributions. Small clusters were evident upon first contact of the platelet with the coated surface, eventually forming throughout the platelet surface; some clusters expanded and coalesced.

### Different collagenous substrates induce different degrees of clustering

**Figure 2:**
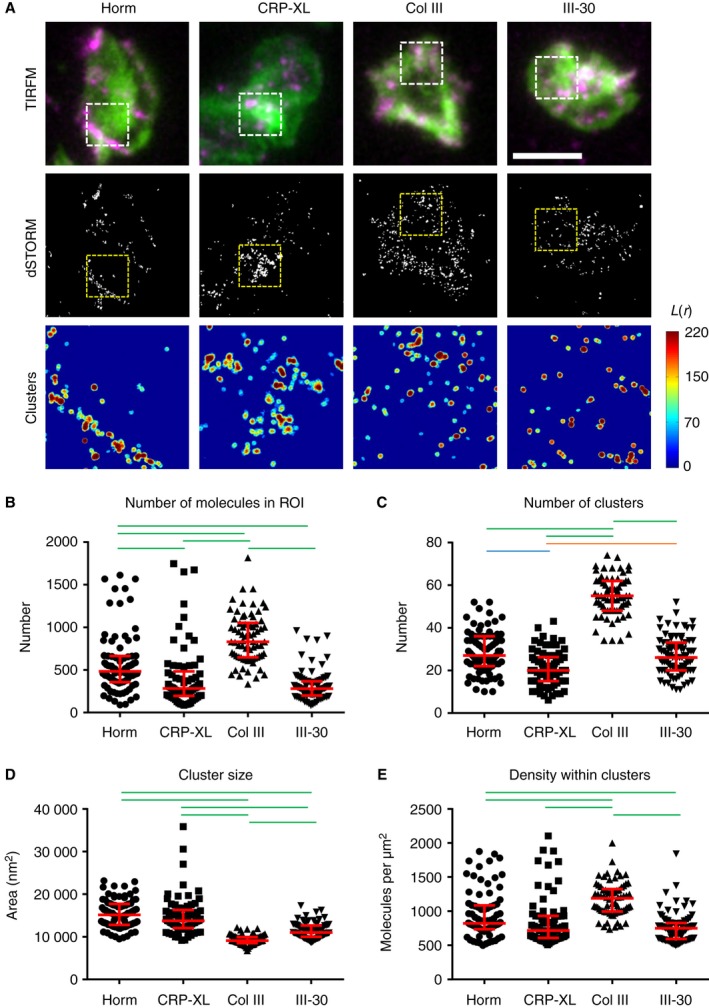
Direct stochastic optical reconstruction microscopy (dSTORM) analysis of glycoprotein VI (GPVI) clustering on collagenous substrates. (A) Platelets spread on the collagenous substrates indicated were labeled for dimeric GPVI with the Alexa Fluor 647‐conjugated Fab 204‐11 (magenta) and F‐actin by use of Alexa Fluor‐488–phalloidin (green), and imaged by total internal reflection fluorescence microscopy (TIRFM) (top row). GPVI was also imaged by dSTORM with the localized points (molecules) shown in the second row. The cluster heat map of the GPVI dSTORM data in the 3 × 3‐μm region of interest (ROI; dashed box in images) is shown in the third row, where red indicates high degrees of clustering. The threshold value of a cluster was set to L(*r*) = 100. (B–E) Quantitative analysis of GPVI dSTORM clustering shows the number of molecules detected in the 3 × 3‐μm ROI (B), the number of clusters in the ROI (C), the size of the clusters (in nm^2^) (D), and the density of the molecules within the clusters (E). All graphs have the median and interquartile range indicated in red. Statistical analysis: non‐parametric Kruskal–Wallis anova with Dunn's multiple comparison. Green lines indicate *P* < 0.001, the blue line indicates *P* < 0.01, and the orange line indicates *P* < 0.05; no line indicates no significance. Scale bar: 5 μm. A total number of ≥ 70 ROIs taken from three or four independent experiments were analyzed for each collagenous substrate. Col III, collagen III; CRP‐XL, cross‐linked collagen‐related peptide; Horm, Horm collagen. [Color figure can be viewed at wileyonlinelibrary.com]

GPVI clustering in fixed platelets spread on the different collagenous substrates was quantified with dSTORM, which has a typical *x–y* resolution of 20–30 nm 24, 25. Widefield TIRFM imaging of the platelets labeled for F‐actin and GPVI dimer showed the same distribution of GPVI as that observed in the live‐cell imaging (Fig. 2A). Cluster analysis of dSTORM data is represented by the heat maps in Fig. 2A. High and low levels of GPVI clustering are shown as red and blue, respectively. All collagenous substrates induced more GPVI clustering than would be expected to occur randomly; however, the cluster distribution depended on the specific substrate. Horm induced high degrees of clustering along the fibers, whereas the other substrates induced clusters that were more evenly distributed throughout the ROI. Quantification showed that there were more GPVI dimers and a higher number of clusters per unit area on platelets spread on Col III than on the other substrates (Fig. 2B,C). These clusters were small (Fig. 2D), but contained the highest density of GPVI dimers (Fig. 2E, median values). Horm induced the next highest number of GPVI dimers per ROI (42% less than Col III), and Horm‐induced clusters were 31% less dense than those on Col III. CRP‐XL and III‐30 were least effective in inducing dimer formation (each with ~ 70% fewer molecules detected in the ROI than with Col III), and cluster densities were also correspondingly reduced as compared with Col III (40% and 37% reduction, respectively). The GPVI dimer clusters formed on Horm and CRP‐XL were not significantly different from each other in size, but were significantly larger than those formed on Col III and III‐30 (Fig. 2D). In summary, all collagenous substrates caused GPVI dimers to cluster, but different numbers of dimers and densities of GPVI within clusters were formed, depending on the nature of the collagenous substrate.

**Figure 3:**
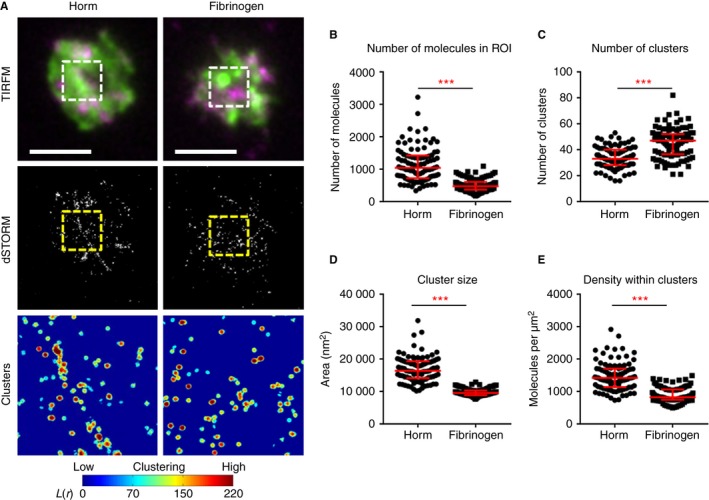
Comparison of glycoprotein VI (GPVI) clustering on Horm collagen (Horm) and fibrinogen. (A) Platelets spread on the Horm or fibrinogen as indicated at the top were labeled for dimeric GPVI with the Alexa Fluor 647‐conjugated Fab 204‐11 (magenta) and F‐actin by the use of Alexa Fluor 488–phalloidin (green), and imaged by total internal reflection fluorescence microscopy (TIRFM). GPVI was also imaged by direct stochastic optical reconstruction microscopy (dSTORM) with the localized points (molecules) shown in the second row. The cluster heat map of the GPVI dSTORM data in the 3 × 3‐μm region of interest (ROI) (dashed box in images) is shown in the third row, where red indicates high degrees of clustering. The threshold value of a cluster was set to L(*r*) = 100. (B–E) Quantitative analysis of GPVI dSTORM clustering shows the number of molecules detected in the 3 × 3‐μm ROI (B), the number of clusters in the ROI (C), the size of the clusters (in nm^2^) (D), and the density of the molecules within the clusters (E). All graphs have the median and interquartile range indicated in red. Statistical analysis: if data passed a normality test, then a *t*‐test was performed (for ‘number of clusters’). If data were not normally distributed, a non‐parametric Mann–Whitney test was used. ***P < 0.001. Scale bar: 5 μm. A total number of ≥ 90 ROIs taken from three independent experiments were analyzed for each substrate. [Color figure can be viewed at wileyonlinelibrary.com]

GPVI dimers were not restricted to the platelet membrane in direct contact with the visible collagen fibers (Figs 1A and 2A). Potential explanations for this are the existence of ‘non‐visible’ microfibers in those areas, to which GPVI dimers can still bind, and/or the fact that fibrinogen or other substances released from adherent platelets may induce cluster formation via outside‐in signaling. To determine whether GPVI clusters can form by collagen‐independent platelet activation, platelets were spread on fibrinogen, and clustering of GPVI dimers was quantified with dSTORM (Fig. 3). GPVI dimer clusters seen on fibrinogen differed from those seen on Horm: the density of dimers in platelets spread on fibrinogen was ~ 50% less than that of dimers in platelets spread on Horm (Fig. 3B); there were many more small clusters on the fibrinogen, and the small clusters were ~ 40% less dense than those formed on Horm (Fig. 3C–E). Although it is possible that GPVI dimer clustering can occur by a GPVI‐independent mechanism, collagen greatly increases the formation of GPVI dimers, which coalesce into larger and denser clusters.

### Relationship between clustered GPVI dimers and other receptors involved in the platelet–collagen interaction

**Figure 4:**
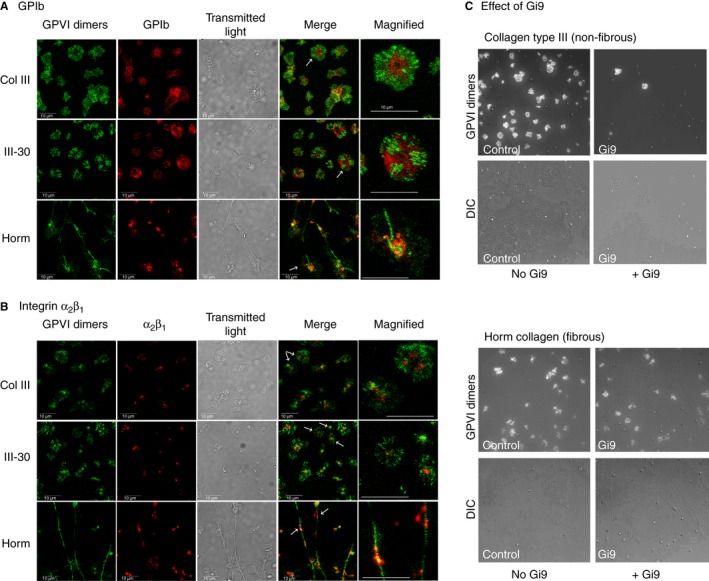
Comparison of distributions of glycoprotein VI (GPVI) dimer clusters, glycoprotein Ib (GPIb) and integrin α_2_β_1_ on adhered platelets, and the effect of inhibiting α_2_β_1_ on cluster formation and adhesion. (A, B) GPIb (set of 15 images in panel A) and α_2_β_1_ (set of 15 images in panel B): confocal images of adhered platelets prelabeled with Alexa Fluor 488‐conjugated 204‐11 Fab (anti‐GPVI‐dimer; 4 μg mL ^−1^, green) and Alexa Fluor 647‐conjugated anti‐human CD42 clone 486805 (anti‐GPIb, 5 μg mL ^−1^, red) or Alexa Fluor 647‐conjugated 16B4 (anti‐α_2_β_1_; 5 μg mL ^−1^, red) and then allowed to adhere on collagen III (Col III)‐coated, III‐30‐coated or Horm collagen (Horm)‐coated dishes. Transmitted light images are included to show the degree of spreading, and magnified images of the platelet(s), indicated by white arrows, are included. The distributions of GPIb were different from those of the GPVI dimers for all three collagenous substrates, and were not associated with the Horm fibers. Integrin α_2_β_1_ bound to the Horm fibers, following a similar pattern as the GPVI dimer clusters, coinciding with GPVI dimer clusters at some points, but not being associated with the lammelipodia of the platelets spread on Col III or III‐30. (C, set of 8 gray scale images, right‐side of the figure) Total internal reflection fluorescence microscopy (TIRFM) images (15‐min time point, upper row in each four‐image group) and differential interference contrast (DIC) images (lower row in each four‐image group). Platelets were prelabeled with Alexa Fluor 488‐conjugated 204‐11 Fab, treated with Gi9 (anti‐α_2_β_1_, 5 μg mL ^−1^; + Gi9) or an equal volume of phosphate‐buffered saline (PBS) (No Gi9), and allowed to adhere on Col III or Horm under TIRFM monitoring for 30 min. Gi9 treatment decreased but did not prevent adhesion and GPVI dimer clustering on fibrous Horm, but little or no adhesion was seen on Col III, even at the 30‐min time point. [Color figure can be viewed at wileyonlinelibrary.com]

The localizations of other receptors involved in the platelet–collagen interaction, i.e. GPIb (Fig. 4A) and integrin α_2_β_1_ (Fig. 4B), were compared with that of GPVI dimer clusters on platelets adhered to collagenous substrates. GPVI dimer clusters formed along Horm fibers, whereas they appeared throughout the lamellipodia of platelets spread on Col III and III‐30. Like GPVI dimer clusters, α_2_β_1_ was found to localize along Horm fibers, but was confined to the cell bodies of platelets adhering to Col III and III‐30; no colocalization with GPVI dimer clusters was observed on the platelet lamellipodia. GPIb (VWF receptor) did not associate with GPVI dimer clusters in the lamellipodia of any of the collagenous substrates tested, remaining in the platelet cell body.

### Contribution of collagen receptor α_2_β_1_ to platelet adhesion and GPVI dimer cluster formation

Platelets labeled with Alexa Fluor 488‐conjugated 204‐11 Fab with or without Gi9 (α_2_β_1_‐blocking antibody), were allowed to settle onto immobilized non‐fibrous Col III or Horm fibers, and monitored with TIRFM. Gi9 treatment prevented platelet adhesion to Col III, but merely decreased the extent of GPVI dimer cluster formation on Horm fibers (Fig. 4C). Gi9 had no effect on platelet adhesion/cluster formation on the GPVI‐specific ligands CRP‐XL and III‐30 (data not shown).

### Effect of Src‐family and Syk kinase inhibition on GPVI cluster formation

**Figure 5:**
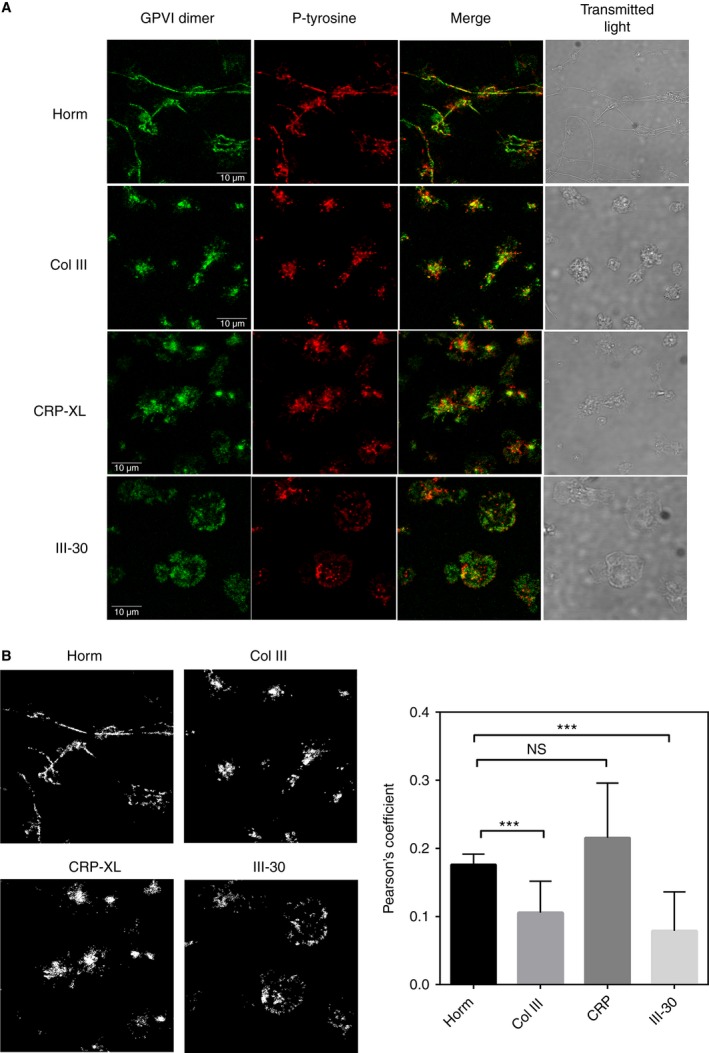
Phospho‐tyrosine (P‐tyrosine) staining and glycoprotein VI (GPVI) dimer clustering. To determine whether signaling reactions may be occurring in the vicinity of GPVI dimer clusters, washed platelets were prelabeled with Alexa Fluor 488‐conjugated 204‐11 Fab, and allowed to adhere on collagenous substrate, and this was followed by formalin fixation, permeabilization with 0.5% Triton/phosphate‐buffered saline, and staining with 4G10 (anti‐P‐tyrosine)/Alexa Fluor 647‐conjugated anti‐mouse Fc. The experiments shown were performed with platelets of one donor, on the same day, and with the same imaging conditions. (A) Confocal images show that, visually, P‐tyrosine (red) was found among, but not necessarily coincident with, the GPVI dimer clusters (green) for platelets adhered to collagen III (Col III), cross‐linked collagen‐related peptide (CRP‐XL), and III‐30. Notably, the P‐tyrosine staining in platelets adhered to Horm collagen (Horm) very closely followed the pattern of GPVI dimer staining, which was mainly confined to the fibers. The morphology of spread platelets was identified by transmitted light. (B) Calculation of colocalization. In the left set of figures, colocalized pixels are presented as a binary threshold mask, calculated with image pro premier v9.2 (Media Cybernetics); they correspond to the respective merged images in (A). The graph on the right shows the calculated Pearson's correlation coefficients for colocalization (mean ± standard deviation), calculated from nine to 11 images, and they are as follows: Horm, 0.176 ± 0.016; Col III, 0.106 ± 0.046; CRP‐XL, 0.216 ± 0.024; and Toolkit peptide III‐30, 0.079 ± 0.057; the Pearson coefficients of the representative images shown in (A) are 0.178, 0.166, 0.179 and 0.074 for Horm, Col III, CRP‐XL and III‐30, respectively. The Pearson's coefficient for Horm was significantly different from those of Col III (***P < 0.001) and III‐30 (***P < 0.001), but not different from that of CRP‐XL (paired *t*‐test, prism v7). These values suggest that only some GPVI dimer clusters are localized with regions of high signaling activity; it is notable that, under the limitations of the resolution afforded by the confocal microscope, both GPVI dimer clustering and signaling can be seen along the Horm fibers. NS, not significant. [Color figure can be viewed at wileyonlinelibrary.com]

**Figure 6:**
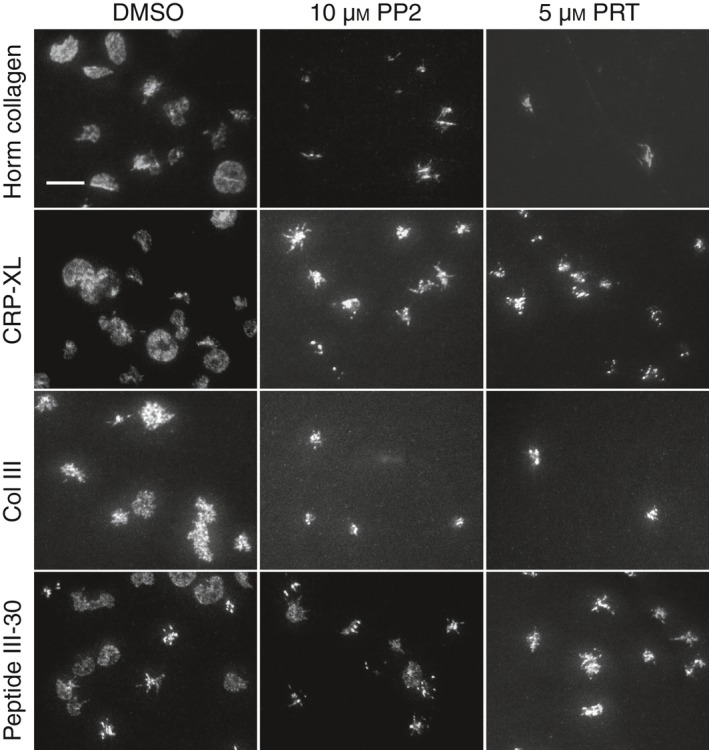
Effect of signaling inhibitors on glycoprotein VI (GPVI) dimer cluster formation in live platelets adhering to immobilized collagenous substrate. Alexa Fluor 488‐conjugated 204‐11‐labeled platelets were reacted with dimethylsulfoxide (DMSO) (vehicle), 10 μm PP2, or PRT‐060318 (5 μm), and their adhesion to immobilized collagenous substrate was then followed by total internal reflection fluorescence microscopy. PRT markedly inhibited platelet adhesion to all substrates, but the platelets that did adhere still showed GPVI dimer cluster formation. The effect of PP2 was weaker but similar to that of PRT. Col III, collagen III; CRP‐XL, cross‐linked collagen‐related peptide.

Confocal images of platelets adhered to Horm, Col III, III‐30 or CRP‐XL showed that areas rich in phosphotyrosine colocalized with some of the GPVI dimer clusters (Fig. 5A), suggesting that signaling was occurring in these regions. The degree of colocalization was analyzed with Pearson's correlation (Fig. 5B, right). To investigate whether protein tyrosine phosphorylation is important in GPVI cluster formation, platelets were treated with the Syk inhibitor PRT‐060318 [2‐((1R,2S)‐2‐aminocyclohexylamino)‐4‐(m‐tolylamino)pyrimidine‐5‐carboxamide] or the Src‐family kinase inhibitor PP2. Both inhibitors, at concentrations that have been demonstrated to inhibit spreading and phosphorylation in human platelets 26, inhibited adhesion on Horm and Col III. Both inhibitors reduced platelet spreading on all collagenous substrates (Fig. S1 shows corresponding DIC images that enable visualization of platelet morphology in the presence and absence of the inhibitors). Despite having a reduction in spreading, discrete GPVI dimer clusters were visible in platelets adhered to CRP‐XL and III‐30 in the presence of PRT‐060318 or PP2 (Fig. 6).

### Effect of disrupting actin dynamics on GPVI dimerization and clustering

**Figure 7:**
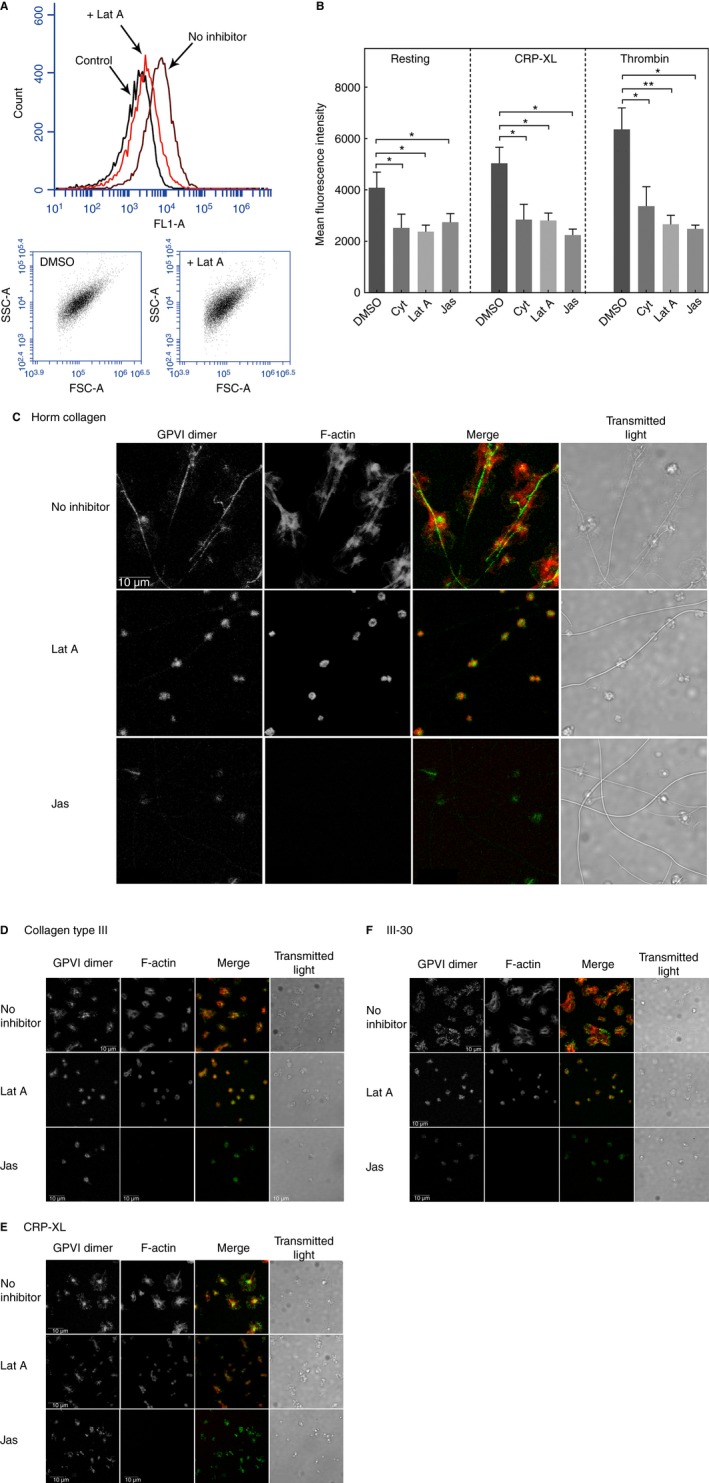
Effect of actin antagonists on glycoprotein VI (GPVI) dimerization and GPVI dimer clustering. (A) Raw flow cytometry data: effects of the actin antagonists latrunculin A (Lat A) and jasplakinolide (Jas) on the levels of dimers in resting and activated washed platelets were determined by flow cytometry (Accuri C6) with fluorescein isothiocyanate (FITC)‐labeled mFab‐F (dimer‐specific). Also shown are a representative histogram and dot‐plots of the control (FITC‐labeled anti‐human Fab), non‐treated resting platelets (no inhibitor, dimethylsulfoxide [DMSO] vehicle at 0.2% final concentration), and Lat A (10 μm)‐treated resting platelets (+ Lat A). There were no obvious differences in the platelet region (SSC‐A versus FL1‐A plot) between the Lat‐A‐treated and non‐treated resting platelets. However, the histogram (upper graph, FLA‐1 versus count) shows that Lat A treatment markedly decreased the level of GPVI dimers, as shown by the clear leftward shift of the Lat A‐treated platelets relative to the untreated resting platelets. (B) Comparison of effects of actin antagonists on GPVI dimer levels in resting and activated platelets. Washed platelets were treated with the vehicle (DMSO) or an actin antagonist at 10 μm: cytochalasin D (Cyt), Lat A, or Jas, and then added with HEPES–Tyrodes buffer (resting), CRP‐XL, or thrombin. The samples were processed for flow cytometry with FITC‐labeled mFab‐F or FITC‐labeled anti‐human Fab (control). Differences among treatment groups were calculated with a paired *t*‐test, by use of prism v7. All tested actin inhibitors decreased GPVI dimer levels (mean fluorescence intensity, mean ± standard error of the mean) in resting platelets (Cyt, Lat A, and Jas, each *P* < 0.05, *n* = 5, as compared with the vehicle alone [0.2% DMSO, final concentration, *n* = 8]), in CRP‐XL‐induced platelets (Cyt, Lat A, and Jas, each *P* < 0.05 [*n* = 5], as compared with 0.2% DMSO), and thrombin‐induced platelets (Cyt and Jas, each *P* < 0.05 [*n* = 5], and Lat A, *P* < 0.005 [*n* = 5], as compared with 0.2% DMSO). (C–F) Confocal images of GPVI dimers and F‐actin in non‐treated and actin‐antagonist‐treated platelets adhered to Horm collagen (C), collagen III (D), CRP‐XL (E), and III‐30 (F). Washed platelets labeled with Alexa Fluor 488‐conjugated 204‐11 Fab (green), with or without treatment with Lat A or Jas, were allowed to adhere on immobilized collagenous substrate, formalin‐fixed, permeabilized, and then stained for F‐actin with Alexa Fluor 647–phalloidin. Lat A (2 μm) and Jas (2 μm) were used at threshold inhibitory concentrations, so that platelet adhesion was not completely prevented. Images were obtained on a confocal microscope, and the following images are shown: gray scale images of GPVI dimer and F‐actin; merged images (green, GPVI dimer clusters; and red, F‐actin); and transmitted light images. Lat A severely inhibited the formation of large GPVI clusters, but a limited number of small clusters could still be observed for platelet adhesion to all tested collagenous substrates. The spreading was clearly inhibited on all of the collagenous substrates. Jas produced more severe inhibition of F‐actin, and no F‐actin staining could be observed in the Jas‐treated platelets. In the Jas‐treated platelets, some green fluorescence could still be observed in the adhered cells, but it is not clear whether this corresponded to small GPVI clusters or was attributable to the higher density of residual GPVI dimers resulting from the much more compact size of the non‐spread platelets. Note that, in spite of the cytoskeleton being severely compromised in the inhibitor‐treated platelets and the total inhibition of spreading, the platelets had residual ability to adhere to the collagenous substrates, possibly because of GPVI dimers, and this was most evident in the platelets adhered along the Horm collagen fibers in the Lat A‐treated or Jas‐treated preparations. [Color figure can be viewed at wileyonlinelibrary.com]

Flow cytometry was used to quantify the effects of drugs that inhibit actin dynamics on GPVI clustering: cytochalasin D and latrunculin A, which are actin polymerization blockers, and jasplakinolide, which is an F‐actin stabilizer. All three inhibitors (10 μm) significantly decreased GPVI dimer levels in resting platelets (Fig. 7A,B). To avoid total abolition of adhesion, we used low‐dose (2 μm) actin antagonist to assess the role of the actin cytoskeleton in clustering of GPVI dimers on Horm, Col III, CRP‐XL, and III‐30 (Figs 7C–D). Low‐dose latrunculin A disrupted the actin cytoskeleton and severely depressed cluster formation, with platelets that did adhere not spreading. Low‐dose jasplakinolide had similar but more severe effects on cluster formation, with no apparent F‐actin staining, and fewer adhered but non‐spread platelets.

## Discussion

GPVI dimers constitutively present on resting platelets represent the collagen‐binding form of this receptor, having over 100‐fold higher affinity for collagen than the monomer 9, 10. The constitutive presence of dimers suggests that a mechanism beyond the formation of dimers may be necessary to initiate signaling through GPVI. One mechanism may be the formation of higher‐order oligomers (clusters), which has been demonstrated to play an important role in the amplification, maintenance and termination of receptor signaling in many cell types 27, 28, including platelets 18, 27. We propose that GPVI clusters may recruit signaling molecules to facilitate platelet activation. To explore this hypothesis, we used complementary imaging methods: TIRFM, dSTORM, and confocal microscopy.

TIRFM enabled the temporal visualization of GPVI cluster formation at the surfaces of platelets spreading on collagenous substrates. After the initial contact of GPVI dimer with the substrate, the number of GPVI dimer clusters increased rapidly. Notably, in platelets adhered to fibrous Horm, discrete clusters formed along the fibers first, and then throughout the surface of the spread platelet (Fig. 1A). This is consistent with GPVI dimers interacting with the surface of the collagen fiber, forming clusters at those points, followed by signaling that induces more cluster formation in regions of the platelet not in direct contact with the visible fiber. A different clustering pattern was observed on the immobilized non‐fibrous collagenous substrates CRP‐XL (Fig. 1B) and III‐30 (Fig. 1D), which exclusively bind GPVI, and soluble Col III (Fig. 1C), which binds GPVI and α_2_β_1_. Immobilized non‐fibrous substrates are randomly orientated triple‐helical molecules, and would be expected to support a different clustering pattern than Horm fibers, which contain a highly organized parallel assembly of triple‐helical tropocollagen molecules within the microfibrils. This structure dictates that GPVI‐binding sites will be distributed on the surface of a fiber at fixed lateral and axial intervals, and suggests that a GPVI dimer would be able to interact with neighboring tropocollagen molecules presenting either separate binding sites or a composite binding site 20.

dSTORM allows single fluorophore molecules to be detected and located with very high spatial precision. Combined with cluster analysis, dSTORM allows quantification of the number of GPVI dimer molecules and the cluster number, size and density in ROIs within a spread platelet. This information permits the relative differences in GPVI clusters to be compared in platelets spread on different substrates. Horm fibers induced a high degree of clustering by virtue of their structure, as outlined above. The spacing and orientation of the two proposed collagen‐binding grooves of GPVI dimer might allow it to bind with increased avidity to sites on adjacent tropocollagen molecules, allowing clustering to occur 20. The ability of immobilized III‐30, CRP‐XL and Col III to bind GPVI will depend on the density and relative orientation of the immobilized substrate helices. CRP‐XL and III‐30 contain only GPVI‐binding motifs, the former having more GPVI‐reactive GPO triplets per molecule, whereas immobilized soluble Col III binds both GPVI and α_2_β_1_, which may support cooperative platelet binding, as motifs for the receptors are located close together. Such differences may explain the variation in cluster density observed between the different collagenous substrates. Indeed, both Horm and Col III had significantly higher GPVI cluster densities than III‐30, and Col III clusters were also significantly denser than those on CRP‐XL, suggesting a measurable role for α_2_β_1_ in these events.

On platelets adhered to non‐fibrous Col III and III‐30, GPIb (Fig. 4A) and α_2_β_1_ (Fig. 4B) did not colocalize with the majority of the GPVI dimer clusters, which are located in the lamellipodia of the spread platelet. However, α_2_β_1_ is indispensable for platelet binding to soluble Col III, as adhesion was severely reduced by the blocking anti‐α_2_β_1_ antibody Gi9. Synergism between GPVI and α_2_β_1_ has also been observed with immobilized model peptides in flowing blood thrombus deposition studies 31. In contrast, Gi9 did not prevent platelet adhesion/cluster formation on fibrous Horm, indicating that GPVI binding to the collagen fiber is much stronger than that to non‐fibrous substrates. These observations are consistent with GPVI being sufficient to support platelet binding to collagen fibers but not to soluble collagen 32. Integrin α_2_β_1_ was also located along Horm fibers in bound platelets, and the magnified confocal images (Fig. 4B) suggest that α_2_β_1_ binds close to some of the GPVI dimer clusters on the fiber. The model proposed by Herr and Farndale 20 suggests that α_2_β_1_ and GPVI might bind ~ 10 nm apart in Col III, and inspection of the collagen I sequence suggests that similar considerations would apply to Horm. Although non‐activated α_2_β_1_ may bind its high‐affinity motif, GFOGER, in Horm fibers 33, this is not essential for GPVI dimer binding and clustering. However, α_2_β_1_ may serve to accelerate platelet adhesion to the collagen fiber 34, thus facilitating further GPVI clustering. Under the present static adhesion conditions, however, the distribution of GPIb differed from that of the GPVI dimer clusters on collagen fibers, although GPIb was observed in the platelet cell body, adjacent to the fiber (Fig. 4A). A mAb against GPIb (SZ2) was reported to block CRP‐XL‐induced platelet aggregation, and GPIb was reported to coprecipitate with GPVI in both resting and thrombin‐stimulated platelets, suggesting an interaction between the two receptors 35. Platelet adhesion to collagen under high shear requires VWF multimers, via GPIb, to tether the platelet to collagen, but our experiments were performed under static conditions in the absence of plasma VWF, so GPIb was less important.

What induces GPVI clustering? One possibility is that platelet activation induced by the initial interaction of GPVI with collagen leads to GPVI oligomerization. However, in spite of severe inhibition of platelet spreading by the Syk inhibitor PRT‐060318, limited cluster formation on all four immobilized collagenous substrates was still observed (Fig. 6, right). The marked inhibition of cluster formation on Col III (which lacks the high‐affinity motif GFOGER) could be attributable to blocking of α_2_β_1_ activation, hence removing the contribution of its high‐affinity form to stabilizing platelet binding to collagen. The Src‐family kinase inhibitor PP2 also inhibited platelet adhesion, but to a lesser extent than PRT‐060318 (Fig. 6, middle). Alternatively, blockade of both Src and Syk might potently inhibit secretion 36, 37, preventing the release of active substances (including fibrinogen), and thus reducing clustering via secondary signaling pathways. These results suggest that, whereas platelet adhesion and spreading is fully dependent on GPVI‐mediated signaling, GPVI cluster formation is only partly so.

Movement of membrane proteins is controlled, in part, by the cytoskeleton in other cells 38. Inhibitors of actin dynamics at 10 μm inhibited GPVI dimer formation in resting platelets, and prevented the GPVI dimer increase in CRP‐XL‐activated or thrombin‐activated platelets (Fig. 7A,B). At 2 μm, a threshold inhibitory concentration, there was severely limited adhesion of non‐spread platelets on all four tested substrates, but there was evidence of residual cluster formation and disturbed actin filament distribution. These results suggest a contribution of the peripheral membrane cytoskeleton to GPVI cluster formation, which is a topic for future investigation.

Although the GPVI dimers on resting platelets are competent to bind collagen, they are not exposed to it in an uninjured vessel, and the low density of GPVI dimers in resting platelets (~ 1500 per platelet) 10 suggests that they may be too far apart to induce efficient signaling; thus, platelets remain inactive, the GPVI dimers requiring both receptor ligation and proximity to activate platelets. However, upon vessel injury, subendothelial collagen fibers are exposed to the bloodstream, and efficient platelet activation is necessary to prevent bleeding. Once a vessel is injured, the binding sites on fibers of collagen types I and III become accessible to the receptors involved in the platelet–collagen interaction, i.e. GPVI, α_2_β_1_, and GPIb. The proximity of GPVI dimer‐binding sites on the fiber surface enables clustering of GPVI dimers, increasing avidity and bringing together the necessary signaling components to initiate signaling and lead to efficient platelet activation and thrombus formation. We examined GPVI‐dependent signaling in the vicinity of GPVI clusters, and whether inhibitors of GPVI‐mediated signaling affected clustering. The proximity of phosphotyrosine to some of the GPVI clusters (Fig. 5), particularly on the Horm fibers, suggests local tyrosine kinase activity. Jamasbi *et al*. 39 recently reported that GPVI‐Fc (Revacept) bound to collagen fibers can be clustered by the addition of anti‐Fc, consistent with dimers binding in close enough proximity in the collagen fiber to allow cluster formation. Activation induces the formation of more dimers, as shown by the increased dimer numbers in CRP‐XL‐activated and thrombin‐activated platelets 10, enabling the formation of even more clusters. Moreover, activation of platelets leads to activation of α_2_β_1_, increasing its affinity for collagen, causing firm adhesion. Our present study suggests that platelets have two layers of activity regulation through GPVI – conversion of monomers to high‐affinity dimers, and clustering of the GPVI dimers. Clustering would serve to increase both avidity for collagen and signaling molecule recruitment, leading to efficient platelet activation during thrombus formation.

## Addendum

N. S. Poulter designed experiments, performed dSTORM imaging, analyzed and interpreted data, created the figures, and wrote the paper. A. Y. Pollitt designed experiments, performed TIRFM, analyzed and interpreted data, created the figures, and wrote the paper. D Owen provided the matlab cluster analysis algorithm and provided expert advice. E. E. Gardiner provided antibodies and critically read the manuscript. R. K. Andrews, H. Shimizu, and D. Ishikawa provided antibodies. D. Bihan synthesized Toolkit peptide III‐30. M. Moroi designed and performed the flow cytometry analyses, created the figures, and interpreted data. R. W. Farndale and S. P. Watson discussed and interpreted data, and critically read the manuscript. S. M. Jung corresponding and senior author, designed and performed experiments (TIRFM, confocal imaging), analyzed and interpreted data, coordinated and wrote the paper, and created the figures.

## Disclosure of Conflict of Interests

The authors state that they have no conflict of interest.

## Supporting information


**Movie S1.** GPVI forms clusters when platelets spread on immobilized collagenous substrates.

**Fig. S1.** DIC images corresponding to the fluorescence images in Fig. 6.
