# Supplementary figures and images for: Clustering of glycoprotein VI (GPVI) dimers upon adhesion to collagen as a mechanism to regulate GPVI signaling in platelets
Source: J Thromb Haemost. 2017 Feb 16;15(3):549–64. doi: 10.1111/jth.13613 (PMC5347898; doi:10.1111/jth.13613)

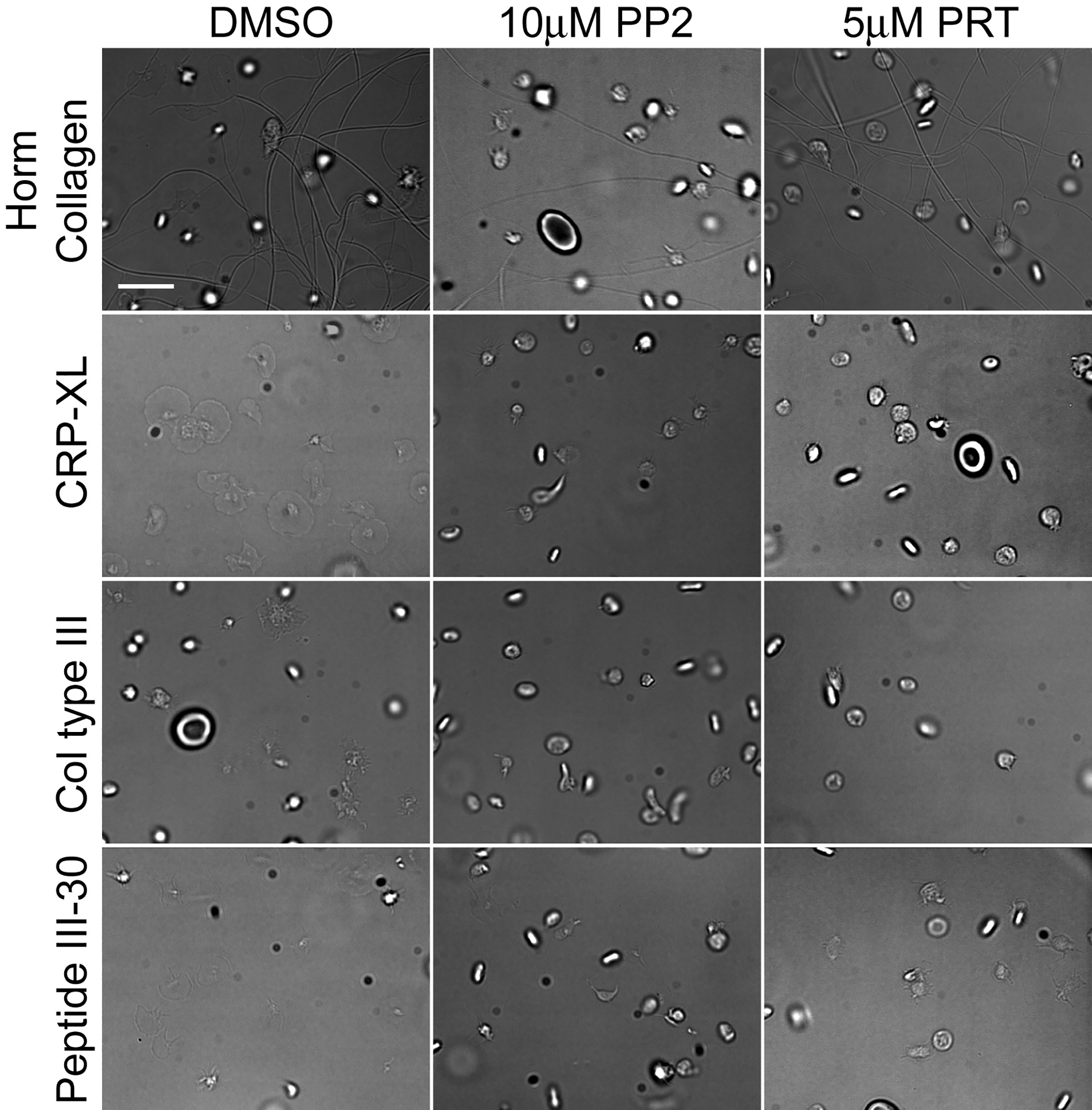

Supplement: Supplementary file 1 — Movie S1. GPVI forms clusters when platelets spread on immobilized collagenous substrates. [file JTH-15-549-s001.tif]
