# Supplementary material for: Clustering of glycoprotein VI (GPVI) dimers upon adhesion to collagen as a mechanism to regulate GPVI signaling in platelets
Source: J Thromb Haemost. 2017 Feb 16;15(3):549–64. doi: 10.1111/jth.13613 (PMC5347898; doi:10.1111/jth.13613)
Supplement: Supplementary file 3 [file JTH-15-549-s003.docx]

Legends for the supplementary materials (Poulter et al., JTH, Article 13613)

Supplementary Movie

GPVI forms clusters when platelets spread on immobilized collagenous substrates. TIRF

microscopy time course of washed human platelets, labelled with Alexa 488 labelled dimerspecific

204-11 Fab, interacting with 10Rg/ml immobilized collagenous substrates at 37oC. (A

(i)) Horm collagen, (B) CRP-XL, (C) peptide III-30 and (D) non-fibrous collagen III. Movies

are a single platelet representative of three independent experiments. Frames were taken

every 5 seconds. Time stamp: seconds. Scale bar: 2 Rm.

Supplemental Fig. 1. DIC images corresponding to the fluorescence images in Figure 6.

Platelets were reacted with DMSO (vehicle), 10 μM PP2, or PRT (5 μM) and then their

adhesion to immobilized collagenous substrates was followed by DIC. PRT markedly

inhibited platelet adhesion to all substrates, but the platelets that did adhere still showed

GPVI-dimer cluster formation. The effect of PP2 was weaker but similar to that of PRT.
